# Evaluating very high energy electron RBE from nanodosimetric pBR322 plasmid DNA damage

**DOI:** 10.1038/s41598-021-82772-6

**Published:** 2021-02-08

**Authors:** K. L. Small, N. T. Henthorn, D. Angal-Kalinin, A. L. Chadwick, E. Santina, A. Aitkenhead, K. J. Kirkby, R. J. Smith, M. Surman, J. Jones, W. Farabolini, R. Corsini, D. Gamba, A. Gilardi, M. J. Merchant, R. M. Jones

**Affiliations:** 1 The University of Manchester, Manchester, UK; 2 The Cockcroft Institute, Daresbury, UK; 3 Division of Cancer Sciences, School of Medical Sciences, Faculty of Biology, Medicine and Health, The University of Manchester, Manchester, UK; 4 The Christie NHS Foundation Trust, Manchester Academic Health Science Centre, Manchester, UK; 5 Christie Medical Physics and Engineering, The Christie NHS Foundation Trust, Manchester, UK; 6 CERN, Geneva, Switzerland; 7 ASTeC, STFC Daresbury Laboratory, Daresbury, Warrington, UK; 8 CEA Saclay, IRFU-DACM, Saclay, France; 9 Federico II, DIETI, University of Napoli, Napoli, Italy

**Keywords:** Radiotherapy, DNA computing, Biological physics, Particle physics

## Abstract

This paper presents the first plasmid DNA irradiations carried out with Very High Energy Electrons (VHEE) over 100–200 MeV at the CLEAR user facility at CERN to determine the Relative Biological Effectiveness (RBE) of VHEE. DNA damage yields were measured in dry and aqueous environments to determine that ~ 99% of total DNA breaks were caused by indirect effects, consistent with other published measurements for protons and photons. Double-Strand Break (DSB) yield was used as the biological endpoint for RBE calculation, with values found to be consistent with established radiotherapy modalities. Similarities in physical damage between VHEE and conventional modalities gives confidence that biological effects of VHEE will also be similar—key for clinical implementation. Damage yields were used as a baseline for track structure simulations of VHEE plasmid irradiation using GEANT4-DNA. Current models for DSB yield have shown reasonable agreement with experimental values. The growing interest in FLASH radiotherapy motivated a study into DSB yield variation with dose rate following VHEE irradiation. No significant variations were observed between conventional and FLASH dose rate irradiations, indicating that no FLASH effect is seen under these conditions.

## Introduction

In the UK, 27% of cancer patients receive radiotherapy as part of their treatment^1^, primarily with 12 MV X-rays though proton therapy treatments are increasing^2^. In the past two decades, developments in high-gradient linear accelerator technology^3,4^ has motivated research into the use of Very High Energy Electrons (VHEE), typcially defined as electrons in the energy range 100–250 MeV, as a radiotherapy modality^5^. By adapting existing high-gradient accelerator technology from linear colliders for high energy particle physics, medical accelerators with accelerating gradients of ~ 100 MV/m could be capable of producing 250 MeV electrons with an accelerator length of 3–4 m.

VHEE radiotherapy has been shown to exhibit potential advantages such as sufficient penetrative range to treat deep-seated tumours, reduced lateral penumbra, relative insensitivity to tissue inhomogeneities^6^ and rapid treatment delivery^7^. This makes VHEE an exciting potential radiotherapy modality and particularly applicable for tumours in highly heterogeneous regions such as the lung. The ability to deliver treatment rapidly makes VHEE a compatible modality for ultra-high dose rate radiotherapy (> 40 Gy/s), referred to as FLASH radiotherapy^8^. At such high dose rates, side effects in normal, healthy tissue have been shown in several in vivo models to be drastically reduced while tumour control rates are maintained^9–11^. Although Bourhis et al.^12^ have presented the first patient treatment by FLASH with favourable outcomes, further understanding of the mechanisms and long-term effects are required before widespread clinical implementation. Combining FLASH therapy with VHEE could provide a potential method to treat tumours in heterogeneous regions while exploiting the benefits of the FLASH effect^13^.

The primary mechanism behind radiotherapy is considered to be DNA damage. Ionising radiation can cause direct or indirect damage to DNA: direct damage is caused by energy deposition from the radiation directly to the DNA structure while indirect damage is caused by free radical attack following the dissociation of water molecules by the radiation, in particular OH^-^ due to their high reaction rate with DNA components^14^. Indirect damage is the main contributor to the total damage following exposure to low Linear Energy Transfer (LET) radiation^15^. The cell is equipped with a complex machinery to attempt to resolve this damage, with Single-Strand Breaks (SSBs) and Double-Strand Breaks (DSBs)^16^ being most difficult to repair. If these breaks are not repaired or are misrepaired, the cell may be unable to function or replicate correctly, potentially leading to cell death or senescence^17^. If physical damage resulting from VHEE irradiation is comparable to damage caused by traditional radiotherapy modalities, this will give confidence that the chemical and biological effects of VHEE are also comparable.

For successful clinical implementation of VHEE, a thorough radiobiological understanding is required along with comparison to well-established radiotherapy modalities through Relative Biological Effectiveness (RBE) to determine if dose prescription for VHEE requires biological augmentation. RBE is defined as the ratio of biological effectiveness of one type of ionizing radiation relative to another, conventionally ^60^Co X-rays, given the same amount of absorbed energy (dose)^18^. This is measured using several endpoints, including DNA damage and, most commonly, cell survival^19^.

This study presents the first pBR322 plasmid irradiations with VHEE beams, over a clinically relevant energy range. Plasmids are ring-like structures of DNA found in bacteria^20^ and were employed to investigate the potential of VHEE to induce DNA damage due to their lack of repair mechanism and ability to study in aqueous and dry environments, allowing the decoupling of direct and indirect damage. Irradiations were carried out at the CERN Linear Electron Accelerator for Research (CLEAR) facility^21,22^. SSB and DSB yields were measured to determine how DNA damage varied with energy and environment. The DSB yields following both dry and aqueous irradiation were used as the biological endpoint for the calculation of VHEE RBE, which was compared to RBE of other radiotherapy modalities. A parameter variation study was then performed for a GEANT4-DNA plasmid irradiation model to determine the parameters which would result in DSB yields which best approximated the experimental data.

The capability of CLEAR to deliver radiation at ultra-high dose rates through ps pulses also allowed the investigation of damage caused to aqueous plasmid samples following irradiation at ultra-high and conventional dose rates. While there is a great deal of research available on ultra-high dose rate irradiation dating back to the 1960s^23^, it typically involves cellular irradiation^24–29^. This study focuses on plasmid irradiation at conventional and ultra-high dose rates to determine the presence of a FLASH effect at the nanoscale. Such an effect would be expected to be a decrease in DNA damage yields however, as plasmid irradiation experiments lack many of the key features that lead to the FLASH mechanism, it was not expected that such a decrease would be observed.

## Results

### Plasmid stability during transportation

For dry plasmid samples, the diluted plasmid solution was transported to CERN and dry samples prepared onsite. For wet samples, a shipment of pBR322 plasmid, held in a solution of 10 mM Tris–HCl and 1 mM EDTA^30,31^, was sent from New England BioLabs to CERN. This was then diluted and wet samples prepared onsite. For both wet and dry samples, control samples were prepared and stored at the Oglesby Cancer Research Centre.

Sham samples were prepared at CERN, mounted on to the sample holder but not directly exposed to the beam. Supercoiled (SC), open-circular (OC) and linear (L) plasmid proportions for control and sham samples were compared to determine the effect of transportation, preparation and indirect radiation exposure. The results are presented in Table 1.

**Table 1:** Comparison of the proportions of supercoiled (SC), open-circular (OC) and linear (L) plasmid in control and sham (unirradiated samples transported to and from CLEAR) plasmid samples.

Sample type	SC	OC	L
Control (dry)	0.867 ± 0.004	0.100 ± 0.003	0.033 ± 0.001
Sham (dry)	0.270 ± 0.013	0.690 ± 0.011	0.038 ± 0.002
Control (wet)	0.877 ± 0.008	0.096 ± 0.006	0.027 ± 0.002
Sham (wet)	0.882 ± 0.008	0.091 ± 0.007	0.027 ± 0.001

Standard error based on three gel electrophoresis repeats and four control samples for each gel.

The plasmid proportion data indicates that transportation in dilute solution did have a significant effect on the dry plasmid samples, with high proportions of open-circular plasmid observed compared to the control samples—indicating a relaxing of the plasmid structure, resulting in SSBs that are not caused by irradiation. Transportation had little effect on the linear plasmid proportion. The effects of transportation were not as severe on the aqueous samples due to transportation in undiluted buffer, which prevented the relaxation of the plasmid structure. Comparison of the dry and wet control samples indicate that the plasmid drying process does not result in significant damage to the plasmid structure.

### Dry sample irradiations

**Figure 1:**
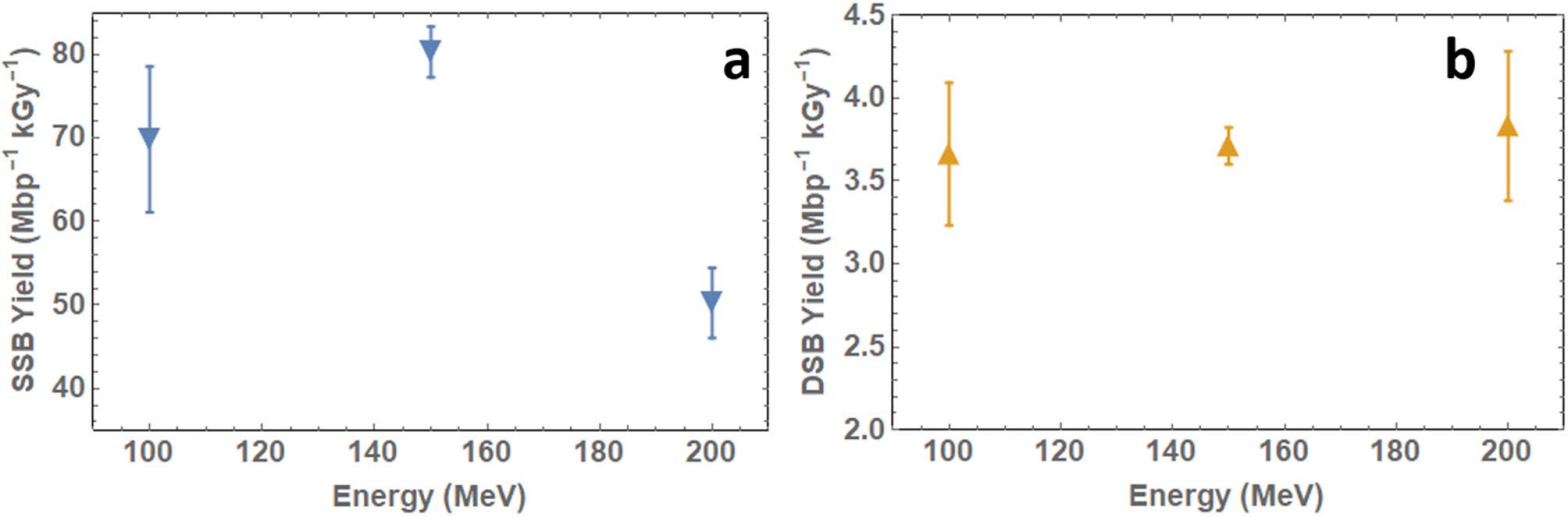
(**a**) Single-strand break yields and (**b**) double-strand break yields for 100–200 MeV electron beam irradiation of dry pBR322 plasmid DNA based on McMahon data fitting^32^.

The damage yields, calculated using the McMahon DNA damage fit^32^, over 100–200 MeV are shown in Fig. 1a, b and in Table 2 below, with standard errors calculated based on three Agarose Gel Electrophoresis (AGE) repeats.

**Table 2:** SSB and DSB yields following dry plasmid irradiation with 100–200 MeV electrons calculated using the McMahon DNA damage fit^32^.

Energy (MeV)	LET (keV/μm)	SSB (Mbp^−1^ kGy^−1^)	DSB (Mbp^−1^ kGy^−1^)
100	0.2202	69.81 ± 8.72	3.66 ± 0.43
150	0.2238	80.30 ± 3.06	3.71 ± 0.11
200	0.2263	50.27 ± 4.19	3.83 ± 0.45

Standard error based on three AGE repeats.

### Wet sample irradiations

**Figure 2:**
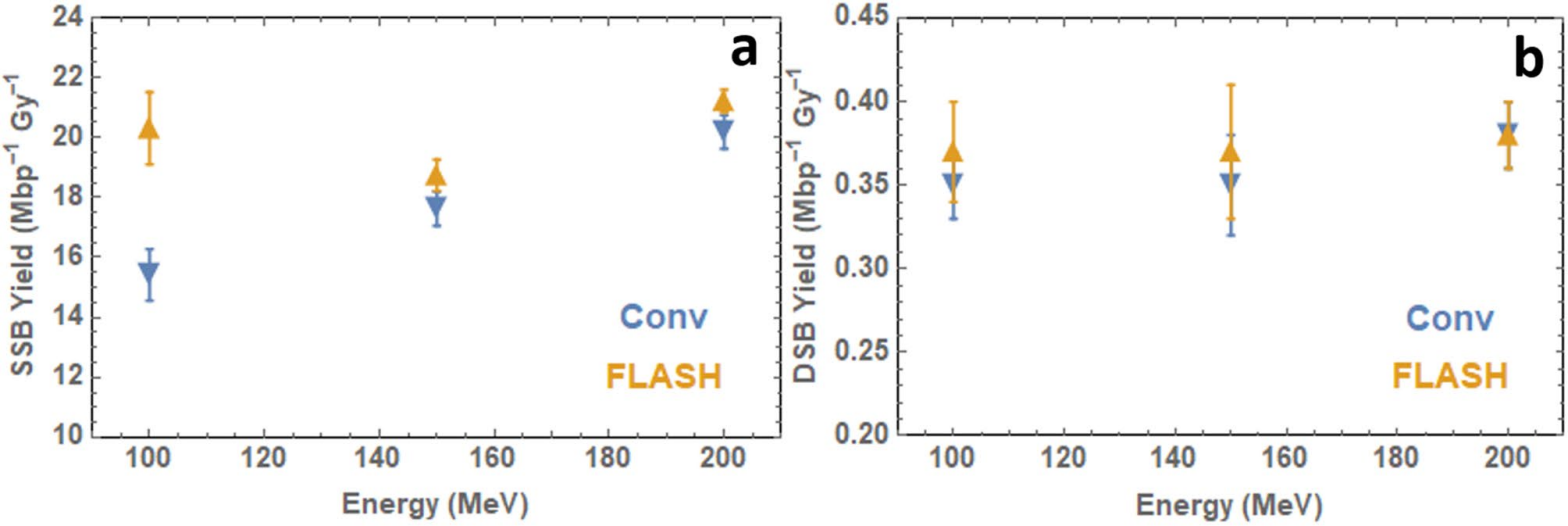
(**a**) Single-strand break yields and (**b**) double-strand break yields for 100–200 MeV electron beam irradiation of wet pBR322 plasmid DNA based on data fitting to the McMahon fit^32^. Plasmids irradiated at Conventional (~ 0.5 Gy/s) and FLASH (> 10^8^ Gy/s) dose rates.

Break yields were compared between plasmid DNA irradiated at a low dose rate (~ 0.5 Gy/s) and at FLASH dose rate (> 10^8^ Gy/s). As discussed in the Introduction, significant differences in DSB yield were not anticipated due to the experimental conditions lacking key features understood to result in a FLASH effect. The plasmid was diluted as appropriate and samples placed in Eppendorf tubes. The SSB and DSB yields based on the McMahon fit are shown in Table 3 and Fig. 2.

**Table 3:** Single- and double-strand break yields following aqueous plasmid irradiation with 100–200 MeV electrons at Conventional and FLASH dose rates, calculated using the McMahon^32^ fit.

Energy (MeV)	VHEE Dose rate			
	Conventional (~ 0.5 Gy s^−1^)		FLASH (> 10^8^ Gy s^−1^)	
	SSB (Mbp^−1^ Gy^−1^)	DSB(Mbp^−1^ Gy^−1^)	SSB (Mbp^−1^ Gy^−1^)	DSB (Mbp^−1^ Gy^−1^)
100	15.42 ± 0.86	0.35 ± 0.02	20.31 ± 1.20	0.37 ± 0.03
150	17.63 ± 0.57	0.35 ± 0.03	18.74 ± 0.52	0.37 ± 0.04
200	20.19 ± 0.56	0.38 ± 0.02	21.22 ± 0.38	0.38 ± 0.02

Standard error based on six agarose gel electrophoresis repeats for 200 MeV and five for 150 and 100 MeV.

To determine the contribution of direct and indirect effects to overall DNA damage, damage yields for dry and aqueous plasmid samples irradiated at Conventional dose rate were compared, with dry yields assumed to be caused by direct effects only and aqueous yields by both direct and indirect effects. The data is shown in Table 4, indicating that, as for protons and photons^31^, indirect effects from VHEE cause > 99% of damage in aqueous solutions with low scavenging capacity.

**Table 4:** Percentage of total DNA damage caused by indirect radiation effects for SSBs and DSBs.

Energy (MeV)	% of SSBs from indirect damage	% of DSBs from indirect damage
100	99.54 ± 0.03	99.0 ± 0.05
150	99.54 ± 0.01	98.9 ± 0.07
200	99.75 ± 0.01	99.0 ± 0.05

### RBE calculation

**Figure 3:**
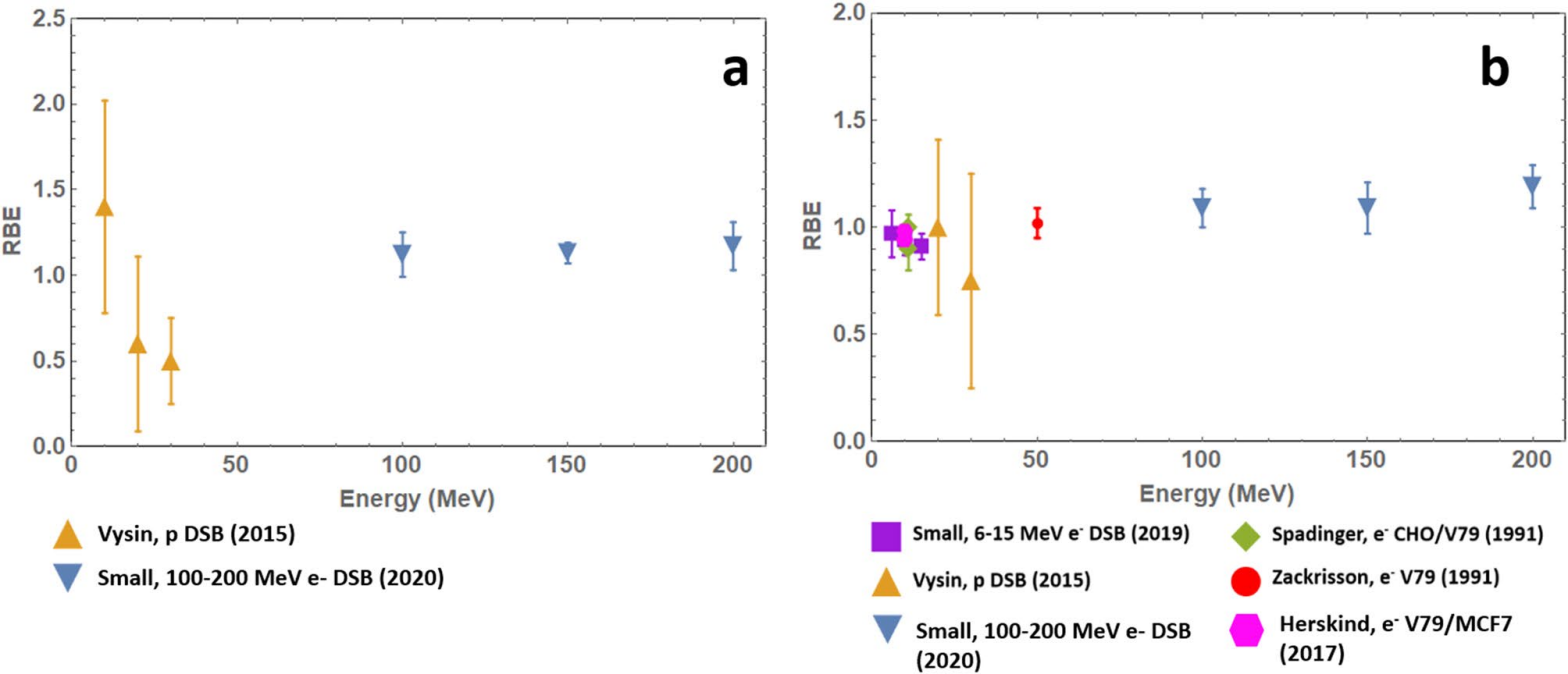
RBE values for (**a**) VHEE and protons with dry plasmid DSB yield as the biological endpoint and (**b**) VHEE, low-energy electrons and protons with wet plasmid DSB yield (Small, Vysin, CLEAR) or cell survival fraction (Herskind, Spadinger, Zackrisson) as the biological endpoint. Experimental data taken from references^35–38^.

DSB yields from dry and aqueous plasmid irradiations were used as the endpoint for calculating VHEE RBE^33^, referred to from this point as RBE_DSB_:

1 $$ \begin{array}{*{20}c} {RBE_{DSB} = {\raise0.7ex\hbox{${\phi_{{e^{ - } }} }$} \!\mathord{\left/ {\vphantom {{\phi_{{e^{ - } }} } {\phi_{\gamma } }}}\right.\kern-\nulldelimiterspace} \!\lower0.7ex\hbox{${\phi_{\gamma } }$}}} \\ \end{array} $$

where ${\phi_{{e^{ - } }}}$ is the DSB yield following plasmid irradiation by electrons and ${\phi_{\gamma}}$ is the DSB yield following irradiation by ^60^Co X-rays—measured as 3.27 ± 0.13 Mbp^−1^ kGy^−1^ for dry and 0.32 ± 0.02 Mbp^−1^ Gy^−1^ for wet plasmids based on experiments performed at the Dalton Nuclear Facility (UK)^34^. RBE_DSB_ values over the energy range 100–200 MeV are given in Table 5 below, along with values for other radiotherapy modalities^35–38^ in Fig. 3 and Table 5.

**Table 5:** RBE for various particle modalities with particle, energy and biological endpoint specified. Standard error of the mean for Small data calculated based on gel repeats.

Particle	Energy (MeV)	RBE	Biological Endpoint
e^-^ (this work, CLEAR)	100	1.12 ± 0.13	Dry plasmid DSB yield
		1.09 ± 0.09	Wet plasmid DSB yield
	150	1.13 ± 0.06	Dry plasmid DSB yield
		1.09 ± 0.12	Wet plasmid DSB yield
	200	1.17 ± 0.14	Dry plasmid DSB yield
		1.19 ± 0.10	Wet plasmid DSB yield
e^-^ (Small)	6	0.97 ± 0.11	Wet plasmid DSB yield
	10	0.94 ± 0.07	Wet plasmid DSB yield
	15	0.91 ± 0.06	Wet plasmid DSB yield
e^-^ (Herskind)	10	0.94 ± 0.02	V79 survival fraction = 0.0003 (rel. to 6 MV X-rays)
		0.98 ± 0.01	MCF7 survival fraction = 0.0003 (rel. to 6 MV X-rays)
e^-^ (Spadinger)	11	1.1 ± 0.08	V79 survival fraction of 0.1 (0–10 Gy)
		1.0 ± 0.04	CHO survival fraction of 0.1 (0–10 Gy)
		1.0 ± 0.06	V79 survival fraction of 0.1 (0–3 Gy)
		0.9 ± 0.1	CHO survival fraction of 0.1 (0–3 Gy)
e^-^ (Zackinsson)	50	1.03 ± 0.08	V79 survival fraction of 0.1 (rel. to 4 MV X-rays)
		1.02 ± 0.07	V79 survival fraction of 0.01 (rel. to 4 MV X-rays)
p (Vysin)	10	1.4 ± 0.62	Dry plasmid DSB yield
	20	1.00 ± 0.41	Wet plasmid DSB yield
		0.6 ± 0.51	Dry plasmid DSB yield
	30	0.75 ± 0.5	Wet plasmid DSB yield
		0.5 ± 0.25	Dry plasmid DSB yield
X-rays (Zackinsson)	20	0.99 ± 0.07	V79 survival fraction of 0.1 (rel. to 4 MV X-rays)
		1.00 ± 0.05	V79 survival fraction of 0.01 (rel. to 4 MV X-rays)
	50	1.14 ± 0.07	V79 survival fraction of 0.1 (rel. to 4 MV X-rays)
		1.12 ± 0.05	V79 survival fraction of 0.01 (rel. to 4 MV X-rays)

RBE data taken or calculated from references^27–31^. All RBE calculations relative to ^60^Co X-ray data unless specified.

### Comparison of GEANT4-DNA and experimental DSB yields

**Figure 4:**
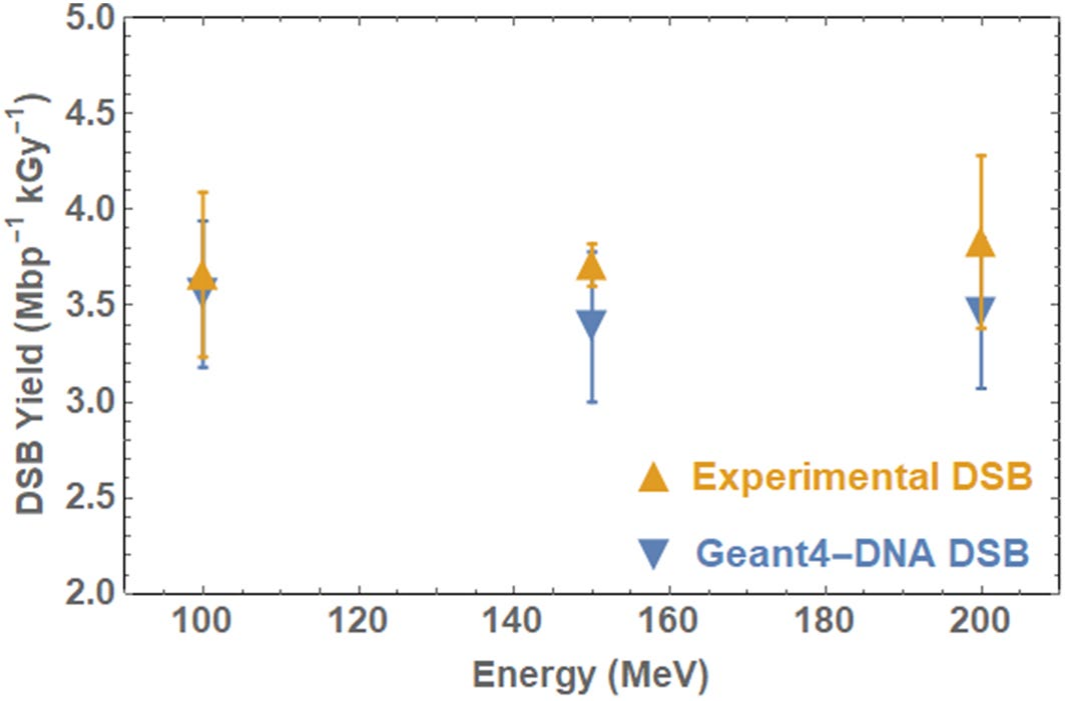
Double-strand break yields for experimental and computational plasmid DNA irradiation with 100–200 MeV electrons. Geant4-DNA simulation performed with half-cylinder DNA geometry with a 10 bp separation defined for DSB induction and damage determined by an energy threshold of 8.22 eV. Standard errors calculated based on 10^3^ repeats.

Experimental DSB data was then compared to GEANT4-DNA simulation data, modelling dry plasmid irradiation with VHEE beams at the same energies as those used at CLEAR. DSB data was chosen as experimental SSB data is likely to be less reliable due to transportation effects. As the parameters based on Henthorn’s earlier work on protons resulted in a significant underestimate in DSB yield compared with experimental data for electrons, a parameter variation study was performed. The geometry, damage scoring method and base pair separation were varied according to similar studies on DNA damage modelling^39–41^ to determine the optimal conditions for simulating plasmid damage with GEANT4-DNA following electron irradiation. This study indicated that use of the energy threshold damage mechanism at 8.22 eV with a separation of 10 bp for DSB induction results in DSB yields which most closely approximated experimental data (Fig. 4), with a more complete set of results available in the Supplementary Material (SF1–SF3).

## Discussion

This work presents the results of the first plasmid irradiation experiments using VHEE at the CLEAR user facility with the aims of investigating the physical and chemical damage caused to DNA following VHEE irradiation. pBR322 plasmids were irradiated in both dry and aqueous environments to investigate the contributions from direct and indirect radiation effects arising from the production of hydroxyl and other radicals. The effect of dose-rate variation was also studied at the DNA damage level, following the rapid resurgence of FLASH radiotherapy and the potential suitability of VHEE as a FLASH radiotherapy modality. DSB yields were then used as the biological endpoint to calculate VHEE RBE.

pBR322 plasmids irradiated in dry and aqueous environments showed little variation in DSB induction over 100–200 MeV, likely due to there being correspondingly little variation in LET (0.220–0.226 keV/μm). Significant variation was observed in the dry SSB yield with electron energy. Relaxing of the supercoiled form of the plasmid to the open circular form was found to have occurred during transportation, resulting in a higher proportion of the plasmid being in an open-circular state before irradiation. For future experiments, it would be recommended that the pBR322 plasmid be shipped directly from New England BioLabs to the facility in the buffer to prevent relaxation and ensure the reliability of SSB measurement.

Comparison of the break yields in aqueous and dry environments allowed the contribution of indirect radiation effects to induced DNA damage to be measured. As anticipated, SSB and DSB yields indicate that indirect effects are the primary cause of DNA damage, causing in excess of 99% of damages. While this seems to contradict the conclusions of Ward et al^42^*.* that indirect effects contribute to ~ 65% of total radiation effects, it is important to note that Ward’s conclusion is based on irradiation of cells, which typically have a scavenging capacity of approximately 200 times that of the diluted aqueous plasmid solution in this study (1 mM Tris). As more radicals are able to cause DNA damage in this plasmid solution, a significantly higher proportion of indirect damage is therefore measured. These results are consistent with Vysin’s measurements of the direct and indirect effect contributions to proton damage of plasmids, but are not directly translatable to cells or tissues due to the significantly lower scavenging capacity.

The effect of dose rate on the irradiation of aqueous plasmids was also investigated, with the aim to determine whether a ‘FLASH’ effect could be observed at the nanoscopic level, in the form of variation in DSB yields following irradiation at conventional or ultra-high dose rates. Figure 2b indicates that there is no statistically significant variation in DSB yield with dose rate. This result is in agreement with our hypothesis that significant DSB variation would not be observed between plasmids irradiated by VHEE at conventional and ultra-high dose rates due to the lack of key experimental features which are the cause of the FLASH effect, notably the use of oxygenated purified water, and room temperature (25ºC) environment. This conclusion is similar to those drawn by other ultra-high dose rate radiobiological studies, though this is understood to be the first study of its kind involving ultra-high dose rate irradiation of plasmid DNA with VHEE. As VHEE is an increasingly popular potential modality for FLASH therapy, a fundamental understanding of the physical effects of FLASH irradiation is crucial.

DSB yields from the dry plasmid irradiation and conventional wet plasmid irradiation experiments were used as the biological endpoint for RBE calculation and compared with RBE of other radiotherapy modalities. VHEE RBE_DSB_ was found to be close to 1 for dry plasmids and 1.1–1.2 for wet plasmids. Comparison with values for clinical electrons suggests that electron RBE may increase with energy.

GEANT4-DNA simulations were carried out in parallel, modelling dry plasmid irradiation with 100–200 MeV electrons to determine the model parameters which could best approximate the experimental conditions. The results of plasmid irradiation simulations indicate that a DNA model built using half-cylinder geometry with damage scored using an energy threshold of 8.22 eV and in which DSBs are induced when two SSBs occur within a separation of 10 base pairs, most closely approximates the CLEAR data. The parameters used here are similar, with small differences, compared to previous in silico studies based on literature-reported experimental data using other radiation modalities including protons, albeit with large variation across datasets. Small differences are likely due to differences between experimental and in silico configuration.

In conclusion, a set of plasmid irradiation experiments was successfully performed at the CLEAR user facility using 100–200 MeV electrons. Little DSB yield variation was observed over the energy (and therefore LET) range. Indirect effects were calculated to contribute > 99% of observed plasmid breaks, consistent with observations for other modalities. No significant variation in damage yield was observed with dose-rate variation, indicating that a FLASH effect is not present, at least for VHEE, at the nanoscale within the plasmid irradiations. Finally, it has been shown, through RBE_DSB_ calculations, that the physical damage caused to DNA by VHEE is similar to that caused by ^60^Co X-rays and low-energy electrons. This provides an indication that more complex biological effects such as cell death could also be similar. This is a key initial pre-clinical step on the way to clinical implementation of VHEE radiotherapy.

## Methods

### Plasmid sample setup

pBR322 plasmid DNA (New England Biolabs) isolated from *E.Coli* (4361 base pairs)^30^ was used in this study. This cloning vector has been extensively used as a plasmid model system in irradiation studies irradiation studies^38,43,44^, allowing direct comparison to be made between this and earlier studies. The plasmid, in solution containing 10 mM Tris–HCl and 1 mM EDTA buffers to prevent degradation during freeze–thaw cycles, was diluted with purified water from 1000 ng/μl to 100 ng/μl. New England BioLabs quotes that ~ 90% of the plasmid is in a supercoiled (undamaged) form. Agarose gel electrophoresis confirmed that between 85 and 90% of the unirradiated plasmid was in this form.

Dry samples were prepared by pipetting 5 μl droplets of 100 ng/μl plasmid DNA directly on to the centre of Permafrost glass microscope slides (25 × 75 × 1 mm^3^, Thermo Fisher Scientific). The droplets were left to dry at room temperature, leaving a thin layer of DNA on the slide. Aqueous samples were held in sealed 1.5 ml Eppendorf tubes, with each tube containing 30 μl of plasmid solution at 100 ng/μl. All samples were stored at -20ºC before and after irradiation.

### Irradiation setup

**Figure 5:**
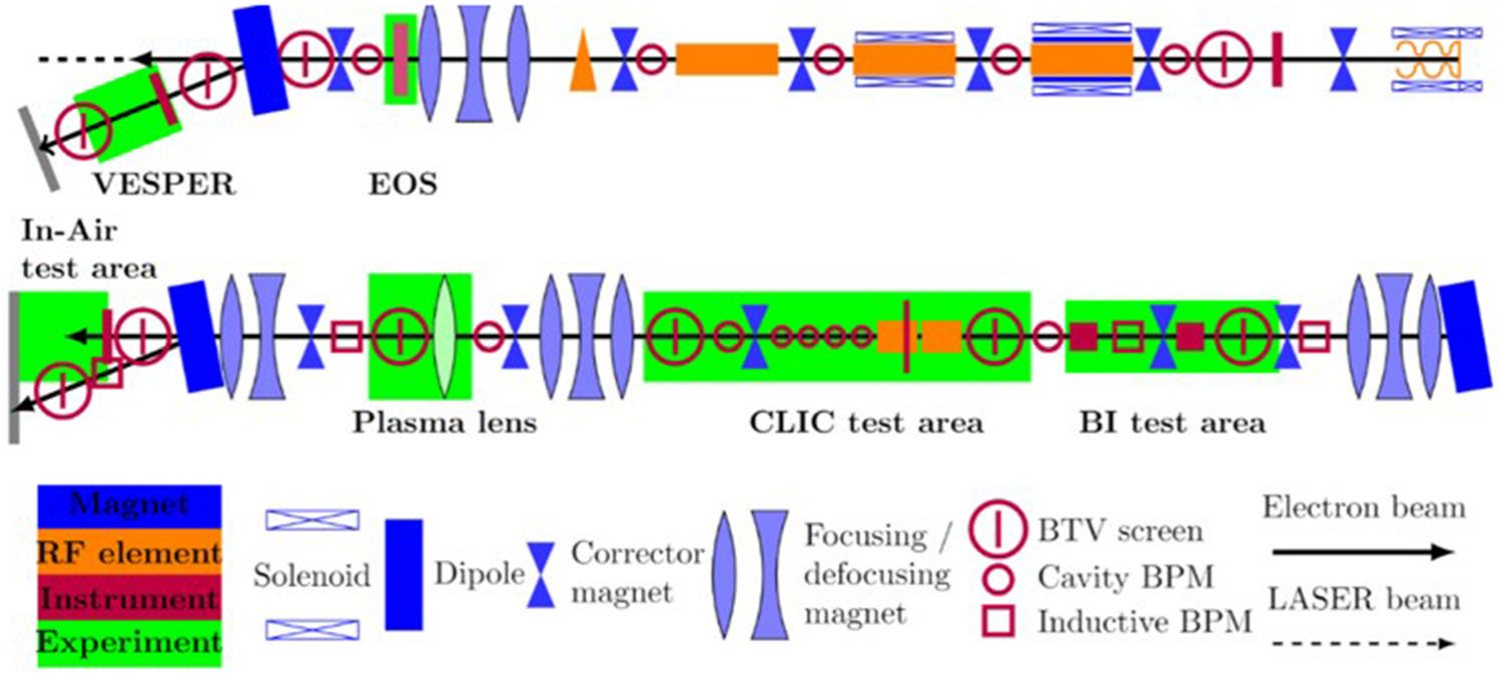
Schematic of the CLEAR beamline and two experimental areas, Figure produced by Kyrre Sjobak^22^ and reproduced here with kind permission from Kyrre Sjobak.

Plasmid irradiations were carried out at the CLEAR user facility (CERN, Geneva), an S-band linear accelerator designed primarily for research and development applications^21,22^. CLEAR is housed in the previous CLEX experimental area and consists of the 25 m CALIFES (*Concept d’Accélérateur Linéare pour Faisceau d’Electron Sonde*) injector, adapted from previous use to test and prove the feasibility of novel two-beam accelerator technology, and a 16 m user beamline. The beamline has two experimental areas, with the in-air beam end area selected for this experiment (see Fig. 5).

The CLEAR user facility was chosen for VHEE plasmid irradiation experiments as it can produce stable high-energy electron beams over an energy range of 60–220 MeV, with readily adaptable beam size, bunch charge and energy—see Table 6 for a full description of beam parameters. CLEAR also has a strong history of VHEE experiments over recent years, including inhomogeneity sensitivity and dosimetry studies from the University of Manchester^6,45,46^.

**Table 6:** CLEAR beam parameters primarily taken from Gamba et al.^21^ and Sjobak et al.^22^ Beam charge and repetition rate updated based on current availability.

Parameter	Value
Beam energy	60–220 MeV
Bunch charge	0.001–10 nC
No. bunches	Variable: 0– > 200
Beam repetition rate	0.833–10 Hz
Bunch repetition rate	1.5 GHz (high bunch charge)
	3 GHz (low bunch charge)
RMS energy spread	< 0.2%
Typical dose per shot	34.29 Gy/shot (200 MeV, dry) 0.69 Gy/shot (200 MeV, wet, Conv)

The experiment was built in the in-air test area, a 1 m space beyond the exit window through which the beam travels before reaching a concrete beam dump. Beam energy and bunch charge were measured using a dipole spectrometer and a Bergoz Integrated Current Transformer respectively. A YAG screen, placed approximately 2 cm behind the plasmid samples measured the beam size, shape and position. Lead bricks provided shielding from secondary X-rays, with a small opening allowing the beam to reach the samples.

**Figure 6:**
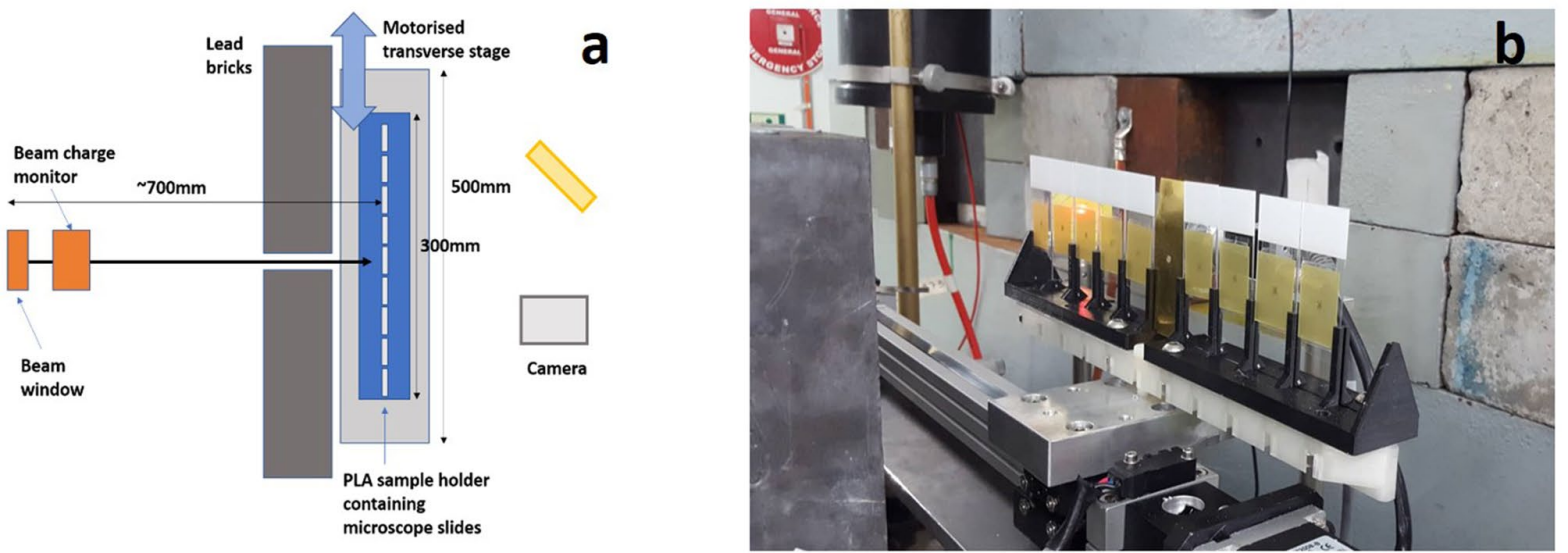
(**a**) Schematic (produced by author K.L.S.) and (**b**) photograph (taken by author K.L.S.) of experimental set-up for irradiation of dry plasmid samples on glass microscope slides. EBT-XD film placed behind samples to show sample coverage by beam.

Dry samples were slotted into a 3D-printed polylactic acid (PLA) slide holder, custom-designed and built at the Cockcroft Institute. This was mounted on to a transversely moving stage placed in front of the beam, presenting each sample to the beam in turn. Dry samples were irradiated at 100, 150 and 200 MeV over a dose range of 1000–6000 Gy with 3 repeats made for each energy and each dose. A schematic and image of the dry experiment setup is shown in Fig. 6.

**Figure 7:**
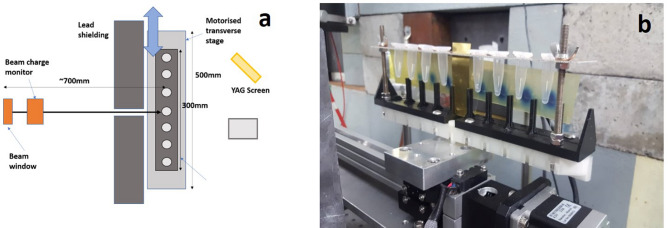
(**a**) Schematic (produced by author K.L.S. and (**b**) photograph (taken by author K.L.S.) of experimental set-up for irradiation of wet plasmid samples in Eppendorf tubes. EBT-XD film placed behind samples to determine dose delivered to samples.

Aqueous samples were held in 1.5 ml sealed Eppendorf tubes and mounted in an aluminium tube holder (see Fig. 7), with irradiation carried out in a similar manner to the dry samples. The samples were again irradiated at 100, 150 and 200 MeV, over a dose range of 0–50 Gy. The difference in dose between dry and aqueous samples arises due to the contribution of direct and indirect effects. To generate observable levels of damage, dry samples must receive a significantly higher dose.

To determine the charge required to deliver the required dose, simulations of the dry and aqueous experimental setups were performed in TOPAS. The dose delivered by 10^7^ electrons was recorded, scaled up to the required dose and the corresponding charge determined. This method has been verified to within a maximum error of 5.26% by Lagzda et al*.*^6,45,46^. For both dry and wet irradiations, EBT-XD film, a radiochromic film commonly used in radiotherapy dosimetry with a dynamic range of 0.1–60 Gy^47^, was placed behind the samples. For the aqueous samples, the film was used to determine the dose delivered to the film, based on 15 MeV electron beam calibration performed at the Christie NHS Foundation Trust using a Varian treatment linac. For the dry samples the film was used to confirm sample coverage only as the dynamic dose range of the film (0–60 Gy) is too low, with dose instead determined from the beam fluence, with beam σ measured using a YAG screen and charge from the beam measured using an ICT. TOPAS simulations revealed a 13–14% difference in dose across the glass slide, which was accounted for in final dose calculations. Further detail is given in the Supplementary material (SF4).

The effect of conventional and FLASH-level dose rates was investigated for aqueous samples to determine if a FLASH effect could be observed at the DNA damage level. The FLASH dose rates were reached by increasing the bunch charge and delivering the radiation in ultra-short (~ ps) single pulses, resulting in dose rates calculated using individual bunch duration in excess of 10^8^ Gy/s.

### Agarose gel electrophoresis (AGE)

Unirradiated plasmid DNA exists in an undamaged, or supercoiled (SC), state. Ionising radiation causes SSBs and DSBs within the DNA, detectable as a change in the plasmid form. Open-circular (OC) plasmid results from a SSB, due to relaxation of the SC DNA. DSBs are detectable as linear (L) forms of plasmid^14^. Determination of plasmid forms was assessed through AGE.

**Figure 8:**
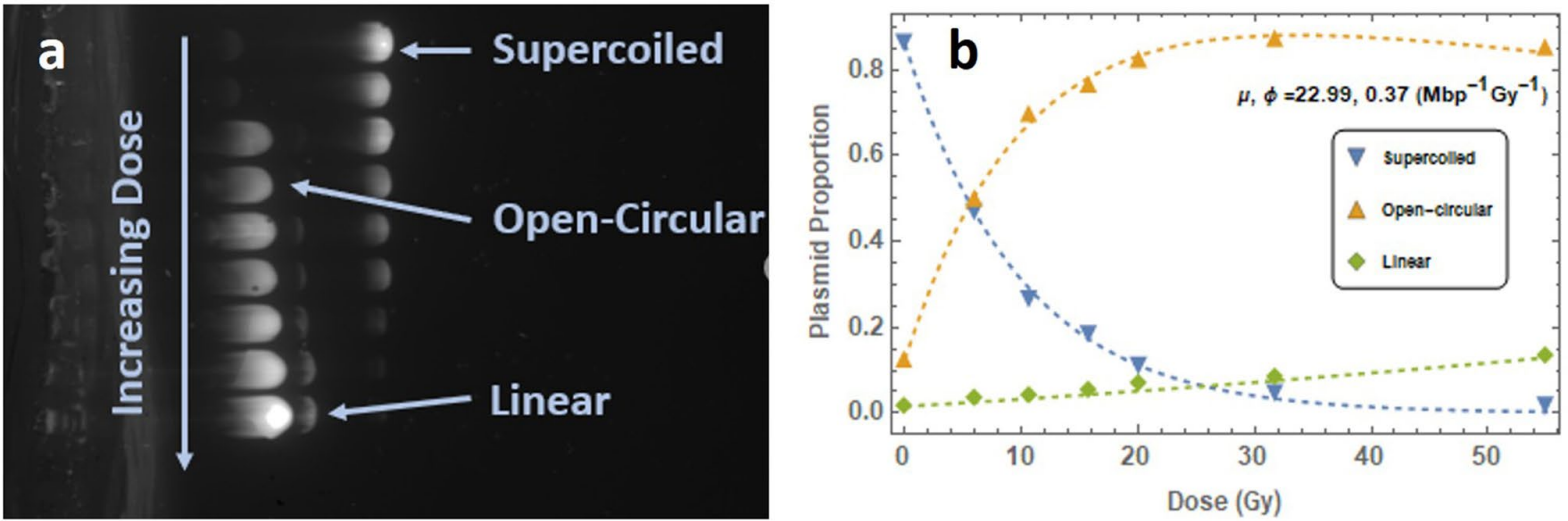
(**a**) Cropped UV image of agarose gel following electrophoresis containing wet plasmids irradiated by 100 MeV electrons. Labelled bands indicate presence of SC, OC and L plasmid forms. Uncropped image shown in SF5. (**b**) SC, OC and L plasmid proportions as a function of dose following integration of band intensities, fitted to Eqs. (2), (3) and (4). Error bars calculated based on five gel repeats.

Dry samples were recovered from the glass slides using 5 μl of purified water and the 30 μl aqueous samples were split into six 5 μl sub-samples. Each sample was mixed with 1 μl gel loading dye and loaded into 5 mm wells in a 1% w/v agarose gel in 1 × TAE buffer stained with 20 μl SYBR Green. The gel was submerged in 0.5 × TAE buffer and a 100 V voltage applied. The gel was run for 120 min or until the samples had migrated 70–80% through the gel. The gels were imaged using a ChemiDoc MP UV imager (BioRad). The plasmid forms appear as distinct bands along the gel (Fig. 8a). Fiji^48^, an open-source image processing package based on ImageJ was used to determine the relative intensities of these bands for each sample, normalised with respect to the most intense band. The proportion of each plasmid form was calculated by integrating the signal over each band.

### Modelling plasmid damage

While several potential fitting methods^49^ are available to determine the yield of SSB and DSBs on DNA, the McMahon^32^ fit was selected for this study based on it being specifically developed for fitting plasmid form proportion data obtained from AGE after irradiation and following a study by Vysin^31^ on the robustness of different fits. The plasmid data are fitted to the following equations:

2 $$ \begin{array}{*{20}c} {SC\left( D \right) = S_{0} e^{{ - \left( {\mu D + \phi D} \right)}} } \\ \end{array} $$

3 $$ \begin{array}{*{20}c} {OC\left( D \right) = e^{ - \phi D} \left[ {e^{{ - \frac{1}{2}\mu^{2} \rho D^{2} }} \left( {S_{0} + C_{0} } \right) - S_{0} e^{ - \mu D} } \right]} \\ \end{array} $$

4 $$ \begin{array}{*{20}c} {L\left( D \right) = 1 - \left( {S_{0} + C_{0} } \right)e^{{ - \left( {\phi D + \frac{1}{2}\mu^{2} \rho D^{2} } \right)}} } \\ \end{array} $$

where $SC\left( D \right)$, $OC\left( D \right)$ and $L\left( D \right)$ are the proportion of supercoiled, open-circular and linear plasmid respectively after irradiation of dose $D$ in Gy, $\mu$ and $\phi$ are the average SSB and DSB yields (Mbp^−1^ Gy^−1^), $S_{0}$ and $C_{0}$ are the supercoiled and open-circular proportions at zero dose and $\rho$ is the probability of a DSB arising due to two SSBs on opposite DNA strands within 10 base pairs. pBR322 plasmid consists of 4361 base pairs, giving $\rho$ = 10/4361.

Figure 8b shows the proportions of SC, OC and L plasmid forms as a function of dose. A least-square error non-linear fit was made to the SC and OC equations to obtain the SSB and DSB yields (μ and φ resepectively). The agreement between the L equation and the linear plasmid data, as observed in Fig. 8b, indicates the efficacy of the McMahon fit.

### Plasmid irradiation simulations with GEANT4-DNA

Plasmid irradiation simulations were carried out to compare DNA damage yields with those obtained from experimental studies. The simulations were carried out using GEANT4-DNA, a module based on the Monte-Carlo particle tracking code GEANT4 (version 10.02-patch01)^50^ designed to model biological damage induced by ionising radiation at the DNA scale^51–54^.

The simulation is based on a plasmid irradiation model designed originally for interaction of proton beams with DNA by Henthorn et al.^55^ pBR322 plasmid DNA, consisting of 4361 base pairs and with radius 236 nm, was built and interaction with monoenergetic electron beams simulated. The GEANT4-DNA default physics list is capable of simulating electrons up to 1 MeV so, to allow use with high energy electrons, the Livermore physics list (G4EmLivermorePhysics) for electrons with energy > 1 MeV was added^56^, with the energy range over which the model functions set to 1–300 MeV.

The irradiation model consists of a circular plasmid placed on a glass slab of density 2.23 g/cm^3^, representing the microscope slide, held within air. The plasmid DNA is built based on a half-cylindrical geometry, as proposed by Charlton et al.^57^. As a simplification, the plasmid geometry is modelled as a closed circle, similar to the approach of McNamara et al.^58^ with the simplified DNA geometry (half-cylinder) as published by Bernal^59^. Each discrete half cylinder is numbered to determine the base pair position on the plasmid. The electron beam is directed perpendicular to the plasmid. Dose to the dry samples was calculated based on the beam fluence, determined using CLEAR beam diagnostics. As the simulation consisted of a single plasmid, the beam radius was scaled down to 300 nm. To ensure the same beam fluence as in the experiment, the number of particles N required to deliver the correct dose was calculated using the following:

5 $$ \begin{array}{*{20}c} {N = \frac{{\pi D\rho r^{2} }}{{10^{9} eL}}} \\ \end{array} $$

where L is the electron LET in units of keV/μm, D is the radiation dose in Gray, r is the beam radius in m and ρ is the DNA density, set at 1407 kg m^−3^.

DNA damage is scored using the same approach as Henthorn^47^ of using two different energy deposition mechanisms to determine the sensitivity of DNA damage yield with energy deposition. The first is based on energy deposition corresponding to a damage probability which increases linearly from 0 to 1 over the energy range 5–37.5 eV, informed by photon and low energy electron DNA damage studies^59^. The second is based on an energy threshold—an energy deposition over this threshold is considered to have caused damage to the DNA. A commonly used value is 17.5 eV^40^, though a review of several studies has shown a range of 8.22–22.5 eV in use^41^.

The damages scored on the DNA volumes are defined as SSBs. DSBs are determined through a clustering algorithm searching for SSBs which have occurred on opposite DNA strands within a specified distance. Output files show the damage data as the number of damages occurring in a single run. The average DSB yields over 10^3^ runs are reported per Mbp per kGy.

## Supplementary Information

Supplementary Information.
