# Supplementary material for: Evaluating very high energy electron RBE from nanodosimetric pBR322 plasmid DNA damage
Source: Sci Rep. 2021 Feb 8;11:3341. doi: 10.1038/s41598-021-82772-6 (PMC7870938; doi:10.1038/s41598-021-82772-6)
Supplement: Supplementary file 1 — Supplementary Information. [file 41598_2021_82772_MOESM1_ESM.docx]

**Evaluating Very High Energy Electron RBE from nanodosimetric pBR322 plasmid DNA damage**

K. L. Small*^1,2^, N. T. Henthorn^3.4^, D. Angal-Kalinin^1,2,7^, A. L. Chadwick^3,4^, E. Santina^3,4^, A. Aitkenhead^3,5^, K. J. Kirkby^3,4^, R. J. Smith^2,7^, M. Surman^2,7^, J. Jones^2,7^, W. Farabolini^6,8^, R. Corsini^6^, D. Gamba^6^, A. Gilardi^6,9^, M. J. Merchant^3,4^, R. M. Jones^1,2^

^1^ The University of Manchester, Manchester, United Kingdom

^2^ The Cockcroft Institute, Daresbury, United Kingdom

^3^ Division of Cancer Sciences, School of Medical Sciences, Faculty of Biology, Medicine and Health, The University of Manchester, United Kingdom

^4^ The Christie NHS Foundation Trust, Manchester Academic Health Science Centre, Manchester, United Kingdom

^5^ Christie Medical Physics and Engineering, The Christie NHS Foundation Trust, Manchester, United Kingdom

^6^ CERN, Geneva, Switzerland

^7^ ASTeC, STFC Daresbury Laboratory, Daresbury, Warrington, United Kingdom

^8^ CEA Saclay, IRFU-DACM, France

^9^ Federico II, DIETI, University of Napoli, Napoli, Italy

E-mail: kristina.small@manchester.ac.uk





Supplementary Figure 1 – Comparison of experimental dry plasmid DSB yield with model DSB yields using parameters taken from Henthorn et al.’s study on proton plasmid damage. Blue triangles – energy range mechanism (5-37.5 eV), SSB separation of 10 base pairs and plasmid built with QuartCyl geometry. Green diamonds – energy range mechanism (5-37.5 eV), SSB separation of 10 bp and plasmid built with HalfCyl geometry. Both significantly underestimate DSB yield compared with experimental data





Supplementary Figure 2 – Results of a parameter variation study in which the energy value for the threshold damage mechanism is varied over the range 8.22-22.5 eV following a review of available literature with plasmid DNA built using HalfCyl geometry. DSB yield is determined at 100, 150 and 200 MeV for each energy value and compared with the experimental data for dry plasmids. 8.22 eV was found to be the value which resulted in DSB yields most closely approximating experimental data





Supplementary Figure 3 – Results of a parameter variation study in which the energy value for the threshold damage mechanism is varied over the range 8.22-22.5 eV following a review of available literature with plasmid DNA built using QuartCyl geometry. DSB yield is determined at 100, 150 and 200 MeV for each energy value and compared with the experimental data for dry plasmids. 8.22 eV was found to be the value which resulted in DSB yields most closely approximating experimental data


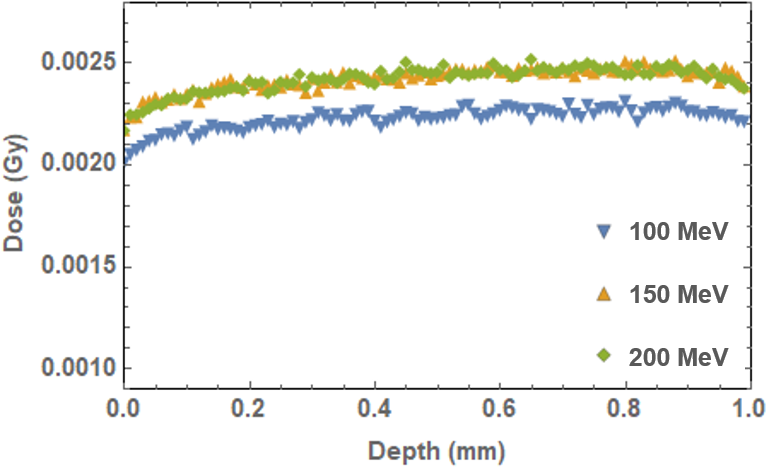


Supplementary Figure 4 – Results of a set of TOPAS simulations to measure variation in dose across a 1mm thick glass microscope slide. Simulation performed with 10^7^ electrons at 100, 150 and 200 MeV. Maximum discrepancy between dose across the slide was determined to be 13.1, 13.5 and 13.9% for 100, 150 and 200 MeV electrons respectively.


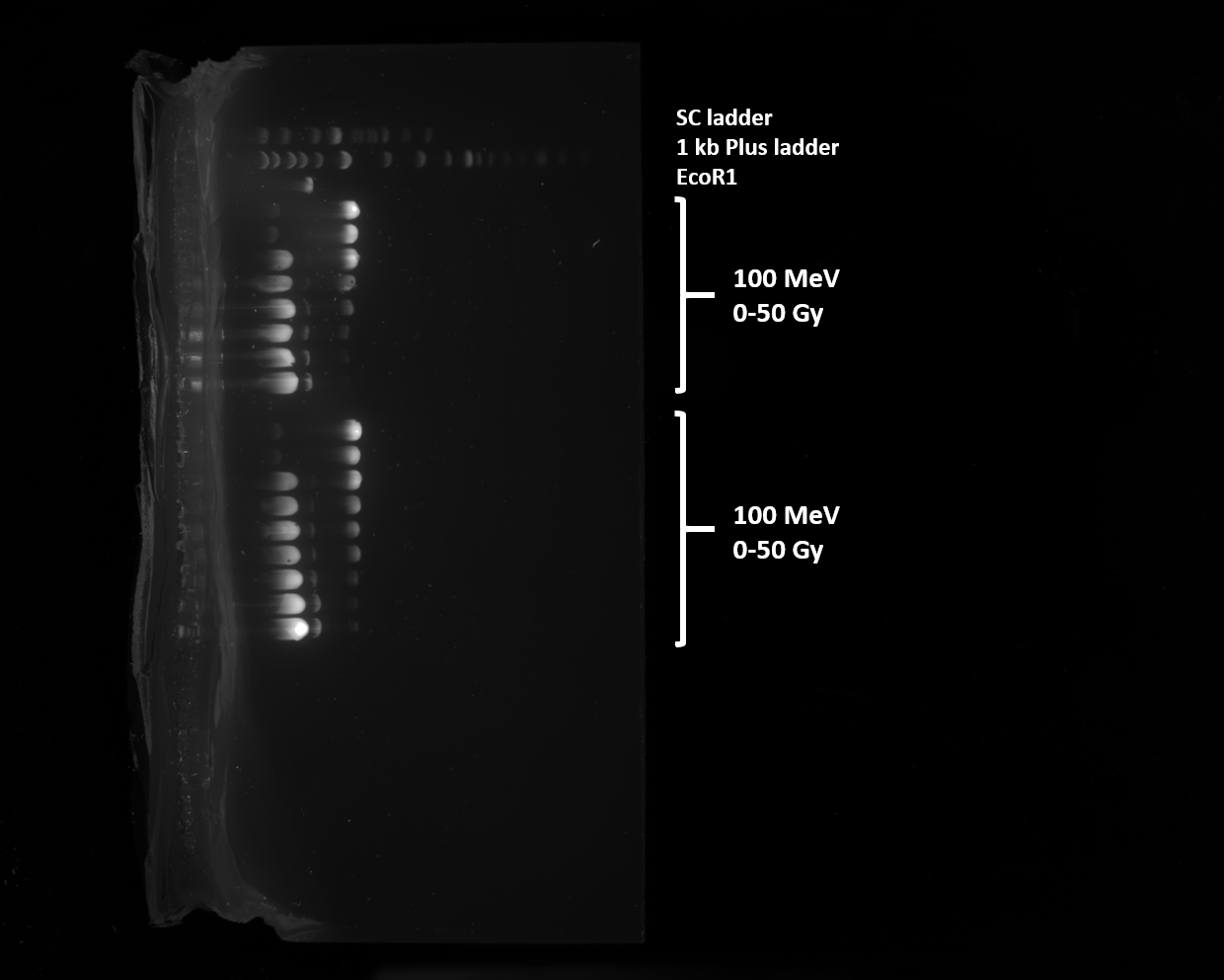


Supplementary Figure 5 – Uncropped version of agarose gel image following electrophoresis of aqueous pBR322 plasmid samples irradiated by 100 MeV electrons over a dose range 0-50 Gy. 2-log and 1 kb Plus size markers and sample containing EcoR1 to indicate position of linear plasmid band have been labelled.
